# Azoxystrobin exposure impairs meiotic maturation by disturbing spindle formation in mouse oocytes

**DOI:** 10.3389/fcell.2022.1053654

**Published:** 2022-12-02

**Authors:** Wen Gao, Chen Zhang, Bichun Li, Jeong Su Oh

**Affiliations:** ^1^ Department of Integrative Biotechnology, Sungkyunkwan University, Suwon, South Korea; ^2^ College of Animal Science and Technology, Yangzhou University, Yangzhou, China; ^3^ RNA Medicine Center, International Institutes of Medicine, Zhejiang University, Hangzhou, Zhejiang, China; ^4^ Biomedical Institute for Convergence at SKKU (BICS), Sungkyunkwan University, Suwon, South Korea

**Keywords:** azoxystrobin, oocyte maturation, spindle formation, MTOC, melatonin

## Abstract

Fungicides are a type of pesticide used to protect plants and crops from pathogenic fungi. Azoxystrobin (AZO), a natural methoxyacrylate derived from strobilurin, is one of the most widely used fungicides in agriculture. AZO exerts its fungicidal activity by inhibiting mitochondrial respiration, but its cytotoxicity to mammalian oocytes has not been studied. In this study, we investigated the effect of AZO exposure on mouse oocyte maturation to elucidate the underlying mechanisms of its possible reproductive toxicity. We found that AZO exposure disturbed meiotic maturation by impairing spindle formation and chromosome alignment, which was associated with decreased microtubule organizing center (MTOC) integrity. Moreover, AZO exposure induced abnormal mitochondrial distribution and increased oxidative stress. The AZO-induced toxicity to oocytes was relieved by melatonin supplementation during meiotic maturation. Therefore, our results suggest that AZO exposure impairs oocyte maturation not only by increasing oxidative stress and mitochondrial dysfunction, but also by decreasing MTOC integrity and subsequent spindle formation and chromosome alignment.

## Introduction

Azoxystrobin (AZO), a natural methoxyacrylate derived from strobilurin, has been one of the most commonly used fungicides in agriculture since 1996, owing to its efficiency and broad-spectrum characteristics in protecting crops (Rodrigues et al., 2013; Abdelraheem et al., 2015). Mechanistically, AZO acts as a specific inhibitor of the ubiquinol oxidizing site (Qo) of mitochondrial respiratory complex III to block electron transfer between cytochrome b and cytochrome c1 in fungal cells. This results in cellular oxidative stress and ATP synthesis failure (Kim et al., 2007). In 2018, the amount of AZO usage was approximately 1,130 tons in the United States (USGS, 2018), and about 10,000 tons were applied to crops in China (Kumar et al., 2020). Since its widespread use, AZO has been prioritized as one of the major chemicals for biomonitoring in many countries due, in large part, to AZO residues in foodstuffs and environmental matrices, including soil, water, and air (Pellizzari et al., 2019; Wang et al., 2021a). In addition to functioning as a pesticide in agriculture, AZO has recently been detected in mold-resistant wallboards at a concentration of 88.5 μg/g, leading to potential pollution in the indoor environment (Cooper et al., 2020).

Given the extensive presence in the environment, studies on AZO exposure effects are numerous. Accumulating evidence suggests that AZO has the potential to induce developmental toxicity in animals. For instance, AZO has been reported to impair cortical migration by increasing reactive oxygen species (ROS) levels and inducing mitochondrial deactivation in mouse embryos (Kang et al., 2021). Also, parental exposure to AZO in zebrafish may cause mortality and developmental malformations in their F1 offspring (Cao et al., 2019). Furthermore, reproductive toxicity of AZO results in reductions in sex hormone production and testis damage in a dose- and time-dependent manner in rats (El-Hak et al., 2022). In addition, a recent study reported that AZO transfers efficiently through the placenta from exposed prenatal mice to offspring (Hu et al., 2022). AZO has been reported to effectively induce esophageal cancer cell apoptosis *via* the mitochondrial pathway and is a potential candidate for drug therapy of esophageal cancer (Shi et al., 2017). Likewise, a positive correlation between AZO exposure and oral cancer suppression *via* inhibition of the mitochondrial respiratory pathway has been well documented (Chen et al., 2020). However, to date, the effects of AZO exposure on oocyte maturation have not been fully elucidated.

Mammalian oocyte maturation is a complex cellular process that is of paramount importance for fertilization and embryo development. After prolonged arrest at the prophase of the first meiosis (MI) which is morphologically identified by a large nucleus called germinal vesicle (GV), oocytes resume meiosis and undergo two consecutive meiotic divisions. After meiotic resumption, spindle microtubules are assembled from microtubule organizing centers (MTOCs) and form a barrel-shaped bipolar spindle with highly organized bundles of microtubules (Mogessie et al., 2018). When spindle microtubules emanated from opposite pole correctly attach to the kinetochores of homologous chromosomes and thereby chromosomes align at the middle of spindle, oocytes enter anaphase and eventually extrude the first polar body (Radford et al., 2017). Oocytes then become arrested again at the metaphase II (MII), awaiting for fertilization. Although many cellular organelles are involved in this process, mitochondria function is of great importance to ensure meiotic maturation and to preserve oocyte quality. Dysfunction of mitochondria could induce the ROS production and eventually led to apoptosis (Kirillova et al., 2021). Moreover, mitochondria have been known to be sensitive to environmental toxicants (Meyer et al., 2013).

Due to the prolonged dormancy, oocytes are susceptible to adverse internal or external factors. Indeed, females exposed to toxic environmental chemicals, such as those found in pesticides, metals, plastics, and food additives, are more vulnerable to reproductive health compromise (Ge et al., 2019). However, the underlying mechanisms of reproductive toxicity have not been thoroughly investigated. In this study, we assessed the effect of AZO on mouse oocyte maturation with the aim of uncovering the underlying mechanisms of its reproductive toxicity.

## Materials and methods

### Animals and reagents

Three- to four-week-old female ICR mice (Koatech, Korea) were used in all experiments. All procedures for mouse care and use were conducted in accordance with the guidelines and approval of the Institutional Animal Care and Use Committee of Sungkyunkwan University (approval ID: SKKUIACUC 2021-09-69-1). Azoxystrobin (131860-33-8, Sigma) and melatonin (73-31-4, Sigma) were dissolved in dimethyl sulfoxide (DMSO) to create stock solutions to be used at dilutions of 0.1% or below.

### Oocyte collection and culture

Ovaries were isolated from mice 46 h–48 h after injection of five international units (IU) of pregnant mare serum gonadotropin (PMSG; HOR-272, Prospec). Mice were humanely euthanized by exposure to CO_2_ and cervical dislocation, and ovaries were placed in M2 medium containing 0.4% bovine serum albumin (BSA; A7906, Sigma) and 100 µM of 3-isobutyl-1-methylxanthine (IBMX) to prevent meiotic maturation. After puncturing antral follicles with a fine needle under a dissecting microscope, fully grown oocytes at the germinal vesicle (GV) stage were collected. For *in vitro* maturation, GV oocytes were cultured for 8 h to allow MⅠ stage progression and 16 h for MⅡ stage progression, during which time oocytes were in IBMX-free M2 medium and covered with mineral oil at 37°C in an atmosphere containing 5% CO_2._

### Azoxystrobin and melatonin treatment

Azoxystrobin (AZO; 131860-33-8, Sigma) were dissolved in dimethyl sulfoxide (DMSO) to create 10 mM stock solutions. The stock was diluted in the M2 medium to obtain a series of working solutions of 1, 5, 8, and 10 µM AZO. The final concentration of DMSO in the medium is 0.1% or below. For ROS scavenging experiment in Figures 5–7, melatonin (73-31-4, Sigma) was dissolved in DMSO to make 10 mM stock solutions and stocks were diluted to 10 µM in the M2 medium.

### Immunofluorescence

After washing three times with PVS (3 mg polyvinyl pyrrolidone/1 mL phosphate-buffered saline), oocytes were fixed in 4% paraformaldehyde at room temperature for 10 min, permeabilized with PVS containing 0.1% Triton X-100 and 0.01% Tween 20 at room temperature for 20 min, and incubated in PVS containing 1.0% BSA at 37°C for 2 h. Oocytes were then incubated overnight at 4°C with anti-α-tubulin (1:1,000; T7451, Sigma), anti-CEP192 (1:200; AR07-PA0001, Young In Frontier), anti-PCNT (1:100; ab4448, Abcam), and anti-p-Aurora A (1:250; 3079, Cell Signaling) antibodies diluted in blocking solution. After washing three times with PVS, oocytes were incubated with Alexa Fluor 488 AffiniPure goat anti-mouse IgG (H + L) (1:500; 115-545-146, Jackson ImmunoResearch) or Alexa Fluor 594 AffiniPure goat anti-rabbit IgG (H + L) (1:500; 111-585-144, Jackson ImmunoResearch) for 2 h at room temperature. Oocytes were then mounted on glass slides, counterstained with DAPI, and examined under a Nikon Eclipse Ti inverted microscope (Nikon).

### Reactive oxygen species detection

DHR123 (D1054, Sigma) was diluted to create a 20 mM stock solution and used at dilutions of 0.1% in M2 medium. Oocytes treated with AZO, MT, or AZO with MT were cultured for 8 h to allow MI progression or for 16 h to allow progression through MII. After washing with M2 medium three times, oocytes were incubated with 20 μM dihydrohodamine-123 (DHR123, D1054, Sigma Aldrich) in M2 medium for 30 min at 37°C in a 5% CO_2_ atmosphere. After washing three times, oocytes were counterstained with DAPI and mounted on glass slides. Oocytes were then observed under a Nikon Eclipse Ti inverted microscope, (Nikon), and the fluorescence was quantified using ImageJ software (National Institutes of Health, NIH).

### Mitochondria detection

Working solution was prepared by diluting 1 µl MitoGreen Indicator with 500 µl Live Cell Staining Buffer (ab112143, Abcam)and 500 µl of M2 medium. Oocytes were incubated with working solution for 30 min at 37°C in a 5% CO_2_ atmosphere. After washing three times with PBS/PVS, fluorescence signals were acquired from live oocytes under a Nikon Eclipse Ti inverted microscope (Nikon). Oocytes were then mounted on glass slides, counterstained with DAPI, and imaged again at large magnification. Images were processed using a Nikon Eclipse Ti inverted microscope and mitochondrial fluorescence was quantified using ImageJ software.

### Expression analysis by reverse transcription-PCR

Total RNAs were extracted from 50 oocytes using the RNeasy Micro Kit (QIAGEN) followed by reverse transcription (RT) using a Sensiscript RT Kit (QIAGEN), according to the manufacturer’s instruction. PCR was performed using the following primers: superoxide dismutase (SOD), 5′-GCA​GGG​AAC​CAT​CCA​CTT-3′ and 5′- TGCCCAGGTCTCCAACAT -3′; glutathione peroxidase (GPX), 5′-GTG​CGA​AGT​GAA​TGG​TGA​GA-3′ and 5′-CTG​GGA​CAG​CAG​GGT​TTC​TA-3′; cytochrome b (CYTB), 5′-AAT​CCA​CTA​AAC​ACC​CCA​CCC-3′ and 5′-GCT​TCG​TTG​CTT​TGA​GGT​ATG​A-3′; *β*-Actin, 5′-GGG​AAA​TCG​TGC​GTG​AC-3′ and 5′-AGG​CTG​GAA​AAG​AGC​CT-3′. The PCR conditions consisted of an initial denaturation at 95°C for 5 min, followed by 40 cycles at 95°C for 30 s, 55°C for 30 s, and 72°C for 30 s and final extension at 72°C for 5 min. The levels of mRNA were normalized to those of *β*-Actin.

### Statistical analysis

All experiments were performed three times. Each experimental group included at least 15 oocytes. The results are expressed as the mean ± SEM from at least three independent experiments unless otherwise stated. One-way ANOVA with Tukey’s post-hoc test was used to analyze the significance of differences between groups (*p < 0.05 = significant difference and **p < 0.01 = extremely significant difference). GraphPad Prism 9 software (GraphPad Software, San Diego, CA, United States) was used for statistical analysis.

## Results

### Azoxystrobin exposure impairs mouse oocyte maturation

**FIGURE 1:**
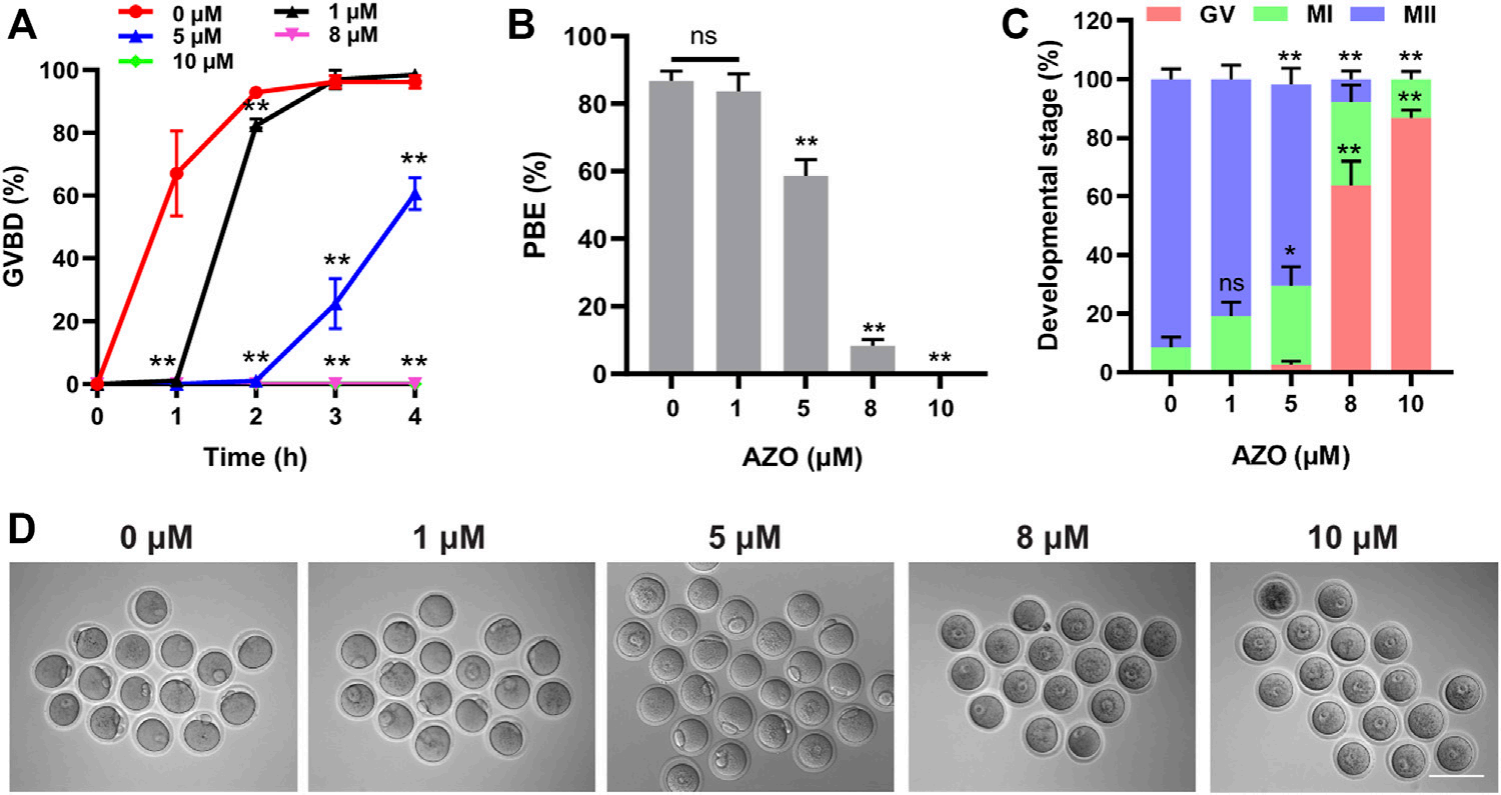
The effect of AZO on oocyte meiotic maturation is dose-dependent. **(A)** Oocytes were treated with different doses of AZO following IBMX release. The percentage of GVBD is expressed as mean ± SEM from three independent experiments. **(B)** After 16 h of culture with AZO treatments using different doses, the percentage of polar body extrusion is expressed as mean ± SEM from three independent experiments. **(C)** Percentage of AZO-treated oocytes at GV, MI, and MII stages. **(D)** Representative images of oocytes at 16 h of culture with different dose-treatments of AZO following IBMX release. Scale bar, 100 μm *p < 0.05, **p < 0.01, ns, not significant.

To determine the potential effect of AZO on oocyte maturation, oocytes at the GV stage were cultured in M2 medium containing different concentrations (0, 1, 5, 8, 10 µM) of AZO for 16 h, and germinal vesicle breakdown (GVBD) and first polar body extrusion (PBE) were examined. As shown in Figure 1A, though a slight delay was observed in GVBD, oocytes treated with 1 μM AZO underwent GVBD. However, 5 µM AZO treatment significantly reduced the GVBD rate, and only about 60% of oocytes underwent GVBD after culturing for 4 h (60.65 ± 5.08). Oocytes exposed to 8–10 µM AZO failed to undergo GVBD from 0 to 4 h. Similarly, in comparison with the control group, 1 µM AZO treatment had no significant effect on polar body extrusion (control, 86.68 ± 2.98; 1 µM AZO, 83.68 ± 5.07). However, 8–10 µM AZO exposure resulted in a sharp decline in PBE rate (8 μM, 8.26 ± 1.87; 10 μM, 0.00 ± 0.00), and most of the oocytes were arrested at the GV stage (8 μM, 63.83 ± 8.13; 10 μM, 86.82 ± 2.57). Notably, 5 µM AZO exposure led to a considerable reduction in PBE rate (58.65 ± 4.81), and about 68.5% oocytes underwent meiosis II (MII) (68.5 ± 5.51) progression (Figures 1B–D). These results suggest that AZO exposure negatively impacts meiotic maturation in mouse oocytes in a dose-dependent manner. Accordingly, we used 5 µM AZO as the working concentration for subsequent experiments.

### Azoxystrobin exposure impairs spindle assembly and chromosome alignment at the MI stage

**FIGURE 2:**
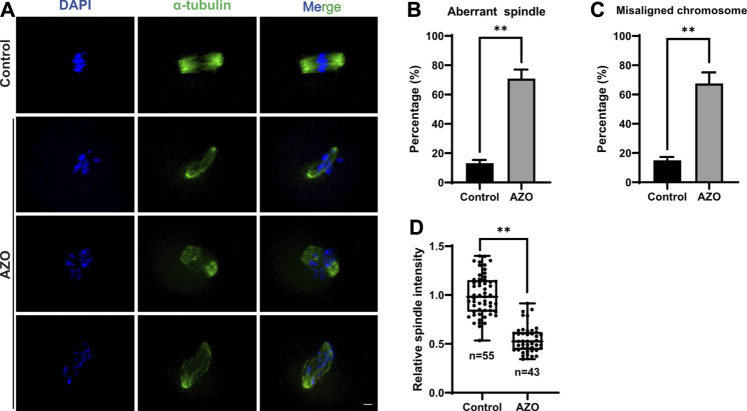
AZO treatment impairs alignment of chromosomes and organization of spindles in oocytes. **(A)** Representative images showing spindle morphologies and chromosome alignment in AZO-treated oocytes and control oocytes. The MI stage oocytes were stained with *α*-Tubulin antibody and DAPI to visualize spindle and chromosomes, respectively. Scale bar, 10 μm. **(B)** The aberrant spindle rate was evident in the control and AZO-treated oocytes at MI stage. **(C)** The aberrant misaligned chromosome was evident in control and AZO-treated oocytes at MI stage. **(D)** Quantification of spindle intensity in control and AZO-treated oocytes at MI stage. Data are expressed as mean ± SEM. **p < 0.01.

Meiotic arrest is usually associated with disorganized cytoskeletal structures (Zhang et al., 2020; Leem et al., 2022). Therefore, we examined the spindle phenotype and chromosome alignment in AZO-exposed oocytes at meiosis I (MI) by immunofluorescence staining. While a typical barrel-shaped spindle and well-aligned chromosomes on the equatorial plate were observed in the control group, the percentages of aberrant spindles (13.16 ± 2.20 vs. 70.86 ± 6.17) and misaligned chromosomes (15.08 ± 2.24 vs. 67.54 ± 7.56) were significantly increased in oocytes after AZO exposure (Figures 2A–C). Moreover, AZO exposure significantly reduced the fluorescence intensity of spindles (1.00 ± 0.03 vs. 0.55 ± 0.02) (Figure 2D). Therefore, these results suggest that AZO exposure impairs proper spindle assembly and chromosome alignment during meiotic maturation in mouse oocytes.

### Azoxystrobin exposure impairs microtubule organizing center assembly during meiotic maturation

**FIGURE 3:**
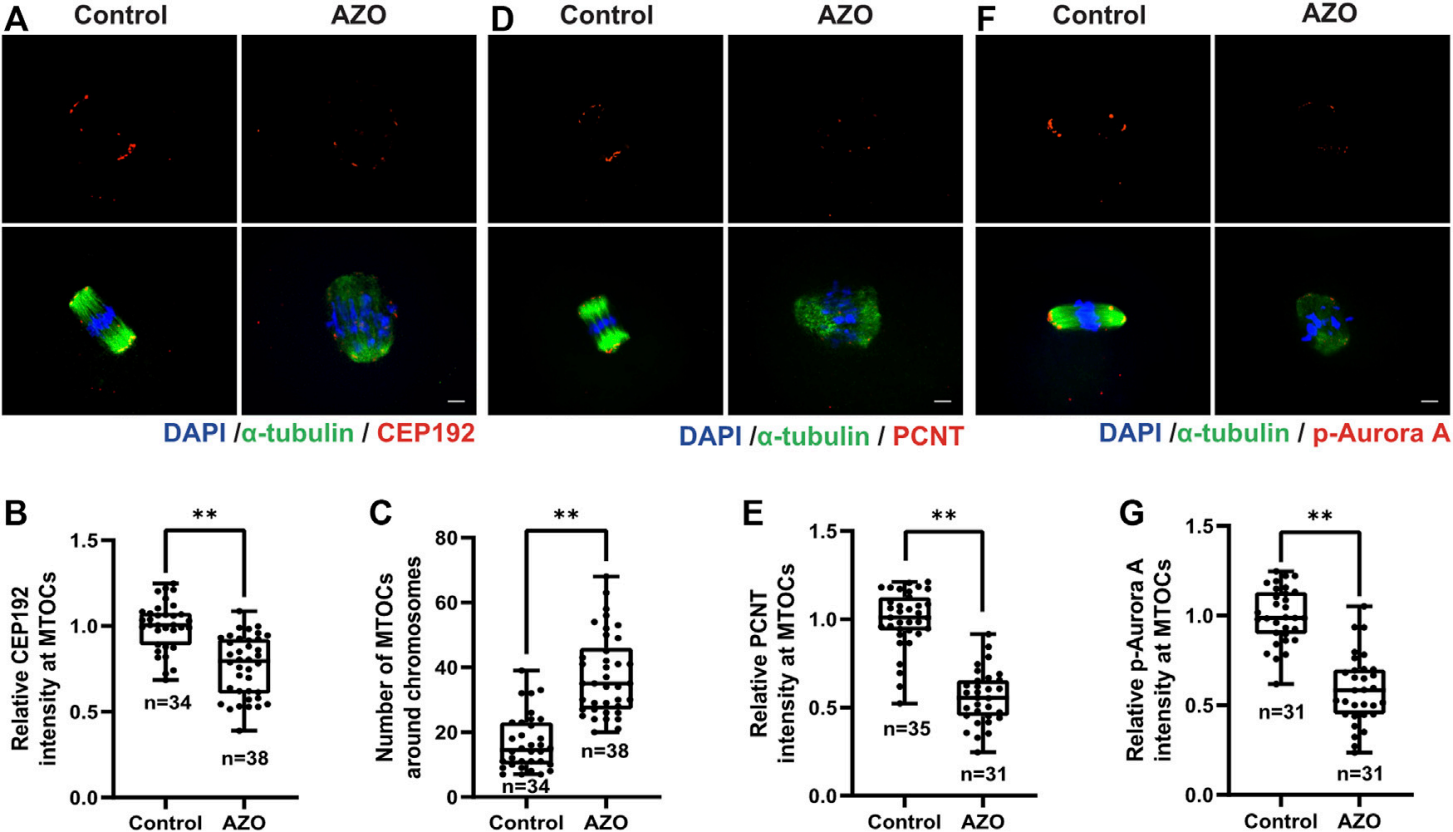
AZO exposure impairs acentriolar MTOC assembly during meiotic maturation. Oocytes were cultured with 5 μM AZO for 8 h and stained with the indicated antibodies and DAPI. **(A,D,F)** Representative images of oocytes labeled with *α*-Tubulin, CEP192, PCNT, and p-Aurora antibodies. **(A)**. Scale bar, 10 μm. **(B,E,G)** The intensities of CEP192, PCNT, and p-Aurora A around MTOCs are quantified. **(C)** The number of MTOCs around chromosomes is quantified using CEP192 fluorescent foci. All data are expressed as mean ± SEM. **p < 0.01.

In many species, the timely migration and accurate positioning of spindles in oocytes proceed through self-organization of multiple acentriolar microtubule organizing centers (MTOCs) (Londoño-Vásquez et al., 2022). To address whether AZO exposure impedes MTOC assembly in the same manner as spindle organization during meiotic maturation, we detected the subcellular localization and intensity of CEP192 as a MTOC marker after AZO exposure (Lee et al., 2018). Compared with the state of CEP192 in control oocytes, AZO-treated oocytes exhibited a disrupted localization and a significant decrease in fluorescence intensity (1.00 ± 0.02 vs. 0.77 ± 0.03) (Figures 3A,B). Moreover, the number of MTOCs was significantly increased in oocytes treated with AZO (16.71 ± 1.45 vs. 37.26 ± 1.12) (Figure 3C). In addition to CEP192, pericentrin (PCNT) and Aurora A play essential roles in accurate spindle assembly and chromosome-microtubule interactions (Baumann et al., 2017; Wang et al., 2021b). Therefore, we examined PCNT and p-Aurora A localization and expression after AZO exposure by immunofluorescence staining. Similar to CEP192, the signal intensity levels of PCNT and p-Aurora A were significantly reduced after AZO treatment (PCNT, 1.00 ± 0.03 vs. 0.56 ± 0.03; p-Aurora A, 1.00 ± 0.03 vs. 0.59 ± 0.03) (Figures 3D–G). These results collectively indicated that the abnormality in spindle organization and chromosome alignment after AZO exposure was related to disruption of the MTOC assembly during meiotic maturation in mouse oocytes.

### Azoxystrobin exposure compromises mitochondrial distribution

**FIGURE 4:**
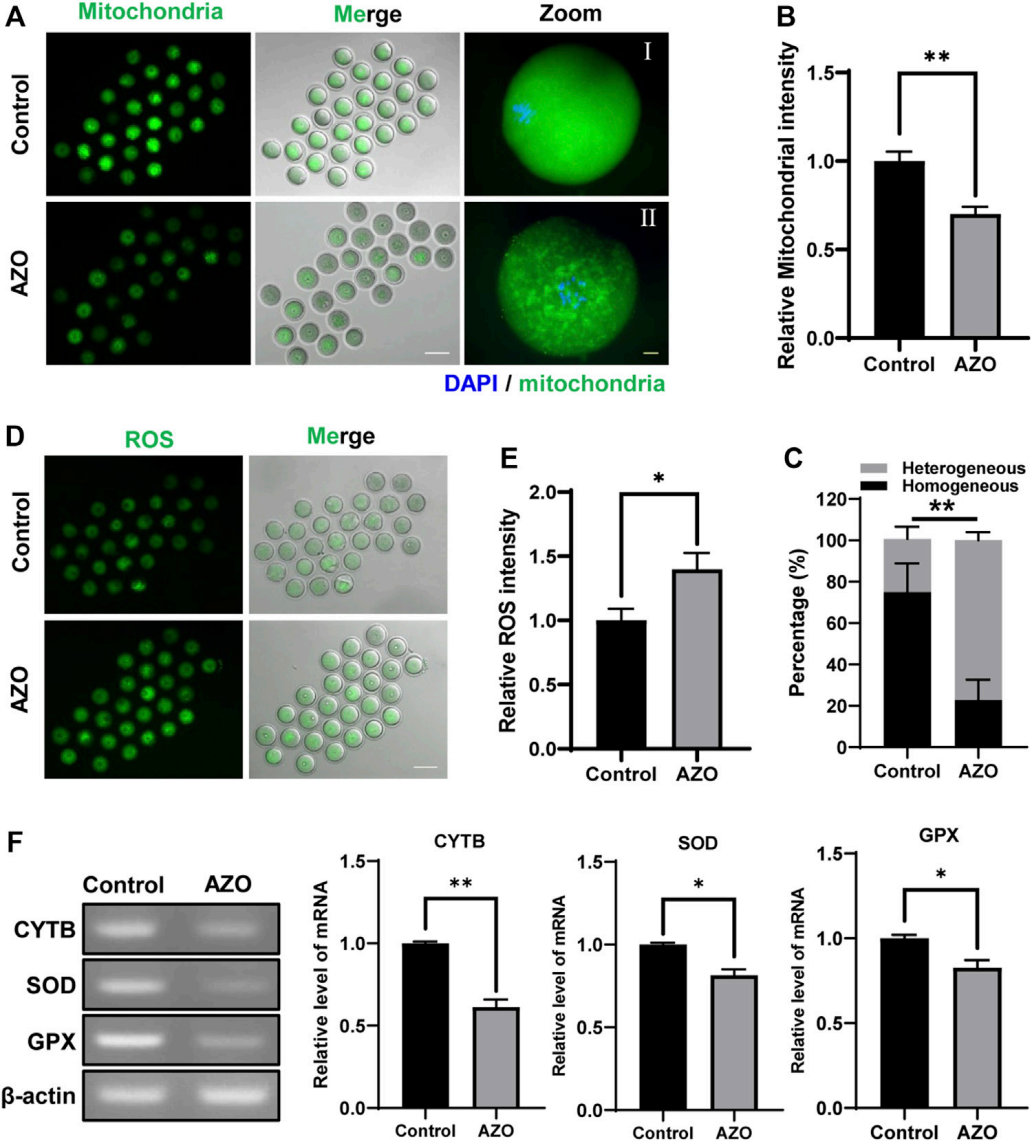
AZO impairs mitochondrial distribution and increases ROS levels. Mitochondria were stained with Mitotracker after 8 h of culture with DMSO (Control) or AZO. **(A)** Representative images of live oocytes stained with Mitotracker. Scale bar, 100 μm (white). Enlarged images of oocyte with homogeneous distribution in the control group and oocyte with heterogeneous distribution in the AZO group. Scale bar, 10 μm (yellow). **(B)** Mitochondrial intensity of oocytes in control and AZO groups is determined. **(C)** Percentage of oocytes with homogeneous or heterogeneous mitochondrial distribution. **(D)** Representative images of live oocytes stained with DHR-123. Scale bar, 100 μm. **(E)** The fluorescent intensity of ROS in control and AZO-treated oocytes. **(F)** Relative mRNA expression of CYTB, SOD, and GPX in control and AZO-treated oocytes. Data are expressed as mean ± SEM from five independent experiments except panel **(F)**. Data in panel **(F)** derived from two independent experiments. *p < 0.05, **p < 0.01.

Since AZO was found to impair mitochondrial function by blocking the electron transfer chain in the inner mitochondrial membrane of microorganisms (Inoue et al., 2012), we determined the effects of AZO on mitochondria in MI-stage oocytes. Our results showed that mitochondria were homogeneously distributed in the cytoplasm in control oocytes (74.93 ± 5.68). However, about 77% of AZO-exposed oocytes exhibited heterogeneous distribution in mitochondria (77.13 ± 2.11) (Figures 4A,C). Moreover, we found that AZO exposure significantly reduced the signal intensity of mitochondria (1.00 ± 0.05 vs. 0.70 ± 0.04) (Figure 4B). Considering that abnormal mitochondrial functioning leads to an increase in reactive oxygen species (ROS), thereby disrupting oocyte maturation through oxidative stress (Kirillova et al., 2021), we next determined the ROS level in MI-stage oocytes exposed to AZO. As expected, AZO exposure significantly increased the ROS levels compared to control oocytes (1.00 ± 0.09 vs. 1.40 ± 0.13) (Figures 4D,E).

To further investigate the effect of AZO on ROS production, we examined the expression of mitochondrial cytochrome b (CYTB) and antioxidant enzymes including superoxide dismutase (SOD) and glutathione peroxidase (GPX) after AZO treatment. We found that AZO treatment significantly decreased mRNA expression of CYTB, SOD, and GPX (Figure 4F). Therefore, our results demonstrated that mitochondrial function was impaired after AZO treatment; and, as a result, oocyte ROS levels were elevated during meiotic maturation.

### Melatonin rescues azoxystrobin-induced meiotic maturation of mouse oocytes

**FIGURE 5:**
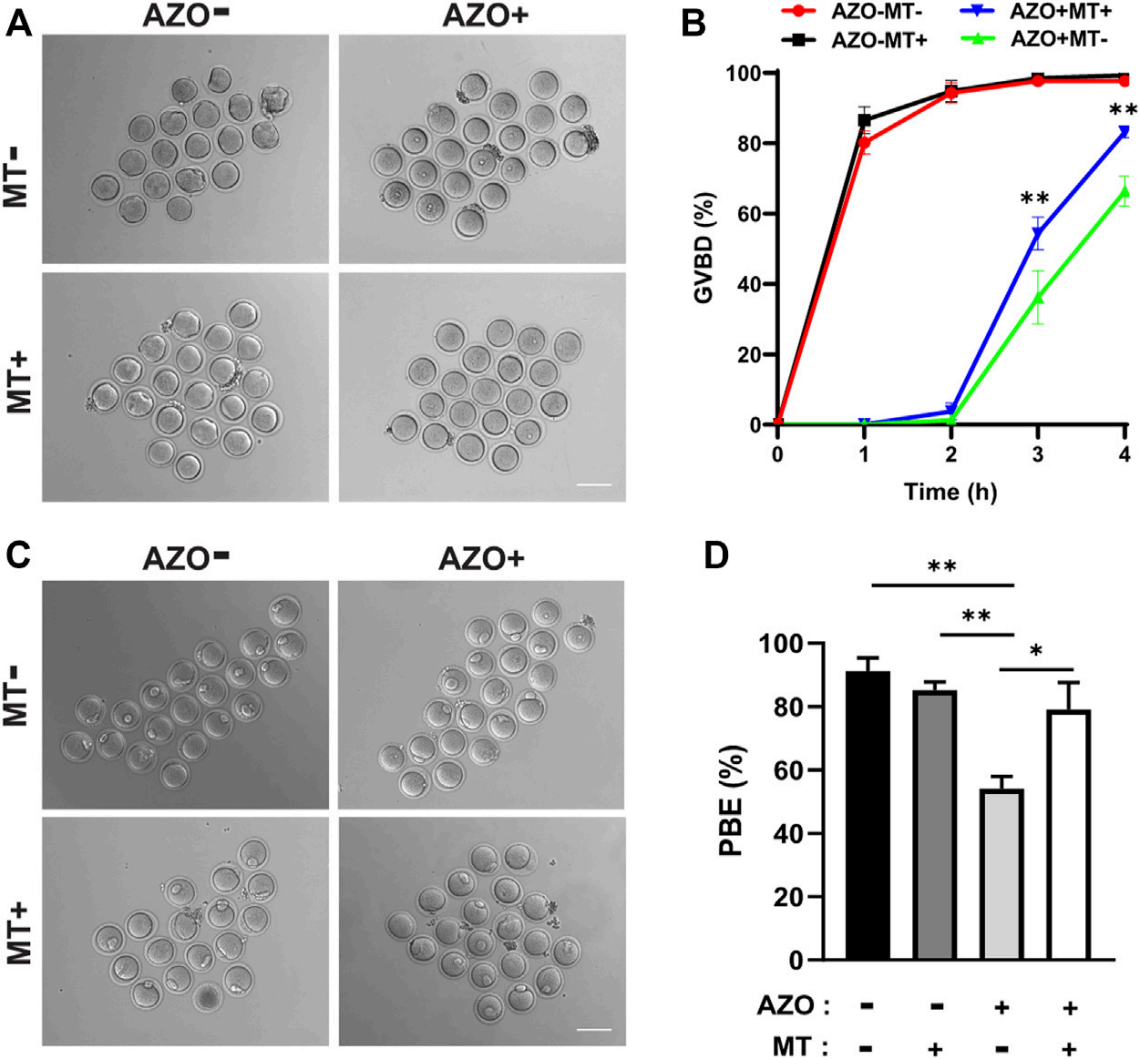
Melatonin rescues PBE impaired by AZO exposure. Oocytes were matured in the absence (−) or presence (+) of AZO and melatonin (MT) during meiotic maturation. **(A)** Representative images of oocytes at 4 h of culture with different treatments following IBMX release. Scale bar, 100 μm. **(B)** The percentage of GVBD is expressed as mean ± SEM from two independent experiments. **(C)** Representative images of oocytes at 16 h of culture with different treatments following IBMX release. Scale bar, 100 μm. **(D)** After 16 h of culture with different treatments, the percentage of polar body extrusion was expressed as mean ± SEM from three independent experiments. *p < 0.05, **p < 0.01.

**FIGURE 6:**
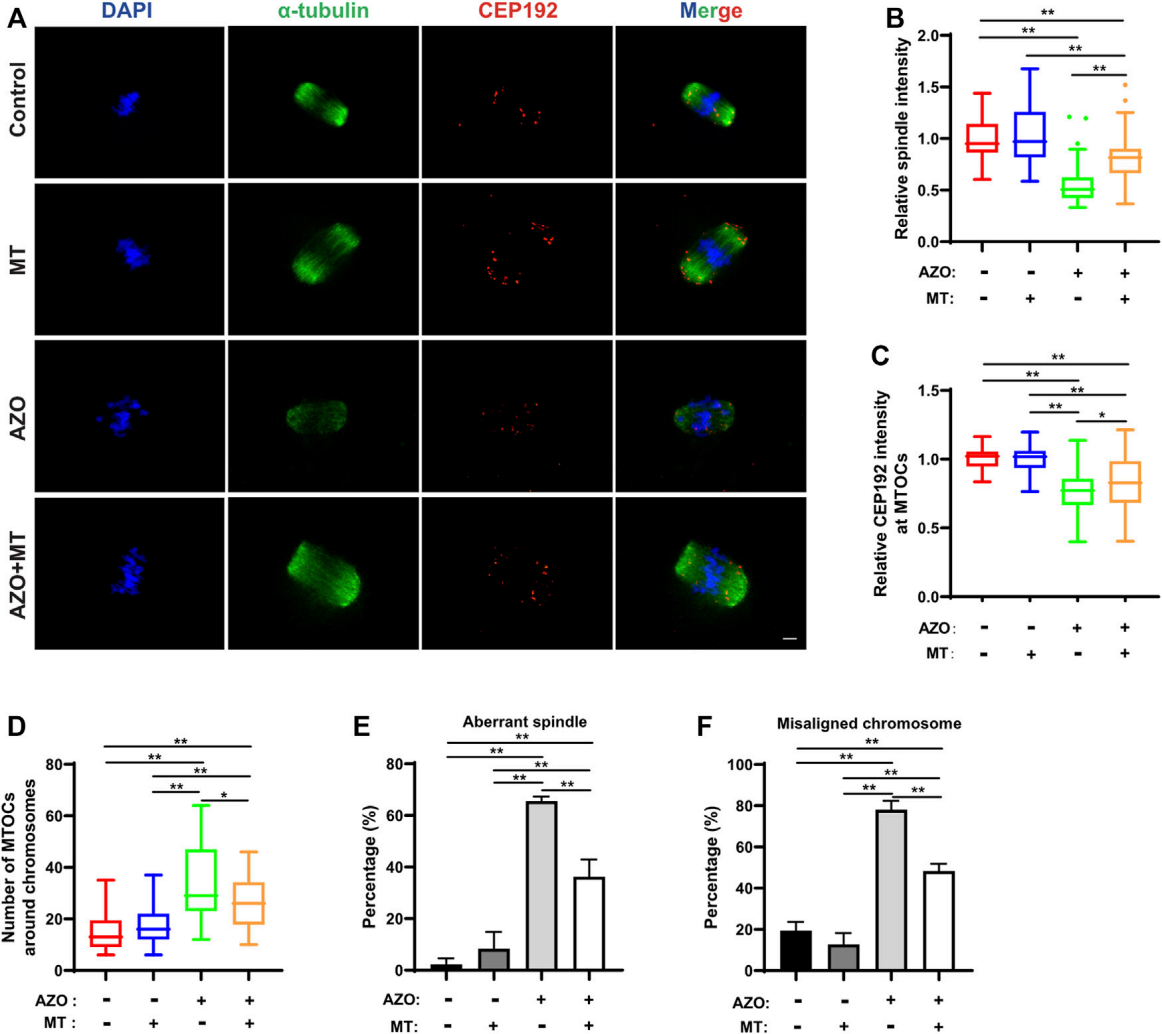
Melatonin rescues AZO-induced impairment of spindle assembly. Oocytes were cultured for 8 h in the absence (−) or presence (+) of AZO and melatonin (MT) during meiotic maturation. **(A)** Representative images of oocytes labeled with *α*-Tubulin and CEP192. Scale bar, 10 μm. **(B,C)** The intensities of spindle and CEP192 around MTOCs are quantified. **(D)** The number of MTOCs around chromosomes is quantified using CEP192 fluorescent foci. **(E)** The aberrant spindle rate was evident in the control and AZO-treated oocytes at MI stage. **(F)** The aberrant misaligned chromosome was evident in control and AZO-treated oocytes at MI stage. All data are expressed as mean ± SEM. *p < 0.05, **p < 0.01.

**FIGURE 7:**
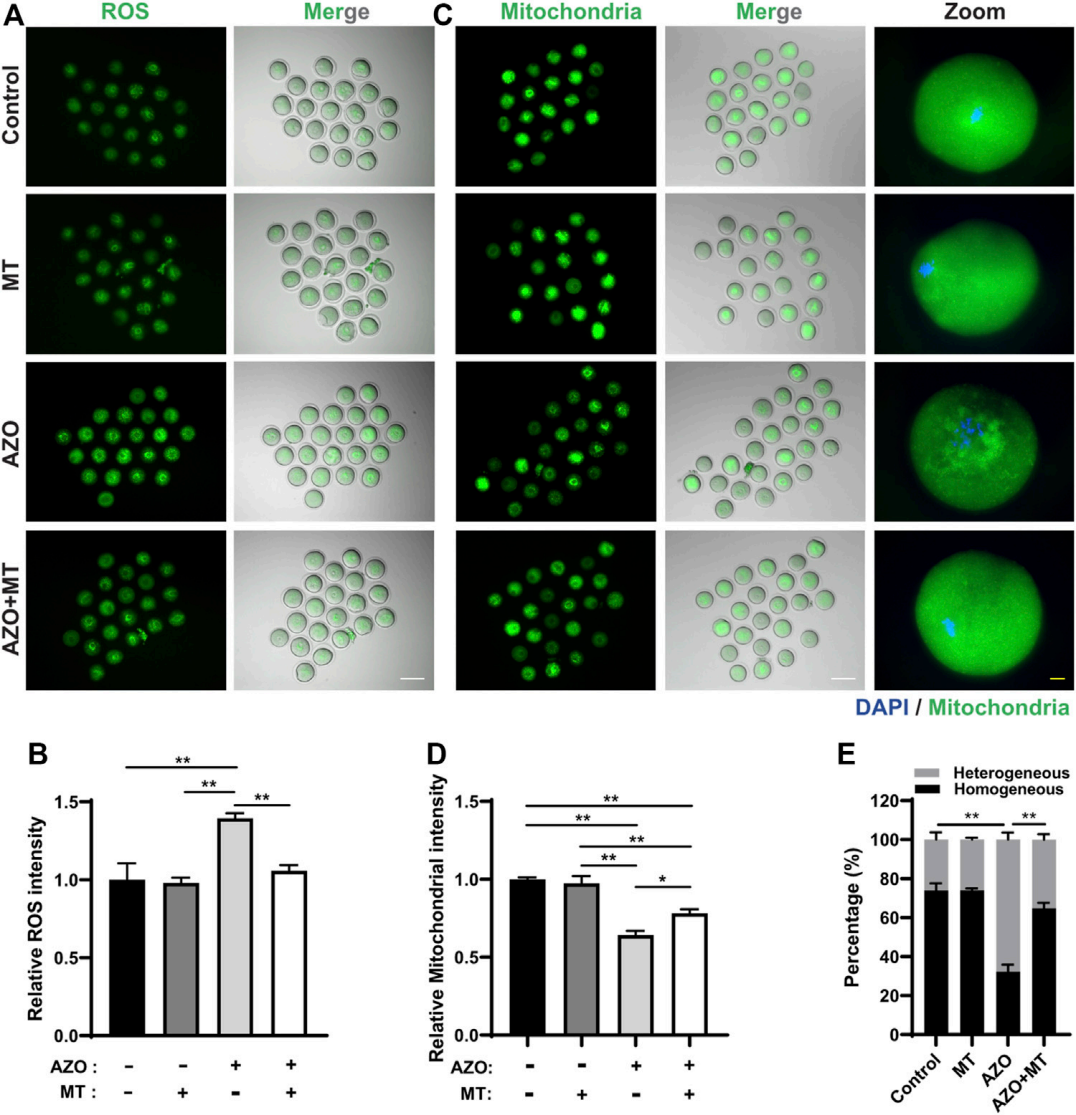
Melatonin protects against oxidative stress and mitochondrial dysfunction induced by azoxystrobin (AZO). Oocytes were cultured for 8 h in the absence (−) or presence (+) of AZO and melatonin (MT) during meiotic maturation. **(A)** Representative images of live oocytes stained with DHR-123. Scale bar, 100 μm. **(B)** ROS intensity of oocytes in control, MT, AZO, and AZO + MT groups are determined. **(C)** Representative images of live oocytes stained with Mitotracker. Scale bar, 100 μm (white). Enlarged images of oocyte with homogeneous distribution in the control, MT, and AZO + MT groups and oocyte with heterogeneous distribution in the AZO group. Scale bar, 10 μm (yellow). **(D)** Mitochondrial intensity of oocytes in control, MT, AZO, and AZO + MT groups are determined. **(E)** Percentage of oocytes with homogeneous or heterogeneous mitochondrial distribution. Data are expressed as mean ± SEM from five independent experiments. *p < 0.05, **p < 0.01.

Given the mitochondrial dysfunction and increased ROS levels in AZO-exposed oocytes, we tested a method designed to reduce ROS levels to determine if oocyte maturation could be rescued. Melatonin (N-acetyl-5-methoxytryptamine) is synthesized and released from the pineal gland and modulates various physiological processes, including female reproduction. It is a strong natural antioxidant and free radical scavenger and has been reported to promote oocyte maturation and embryo development through its antioxidant effects (Galano et al., 2011; Tamura et al., 2012). Therefore, we chose melatonin to test the effect of ROS scavenging on AZO-induced oocyte maturation suppression. GV oocytes were randomly assigned to four groups: the control group (Control), AZO-treated group (AZO), melatonin-exposed group (MT), and AZO-MT-exposed group (AZO + MT). Melatonin was diluted in 5 µM AZO medium to a final concentration of 10 µM. Compared to the exclusive treatment with AZO, addition of melatonin significantly increased the GVBD rate and PBE rate in oocytes cultured for 4 and 16 h, respectively (GVBD, 66.40 ± 4.29 vs. 83.15 ± 1.69; PBE, 54.17 ± 3.73 vs. 79.12 ± 8.43) (Figures 5A–D). Moreover, the intensity of spindle microtubules (0.57 ± 0.03 vs. 0.81 ± 0.03) and CEP192 (0.77 ± 0.02 vs. 0.84 ± 0.03) at MTOCs and the number of MTOCs (34.70 ± 2.29 vs. 27.38 ± 2.11) were restored after melatonin treatment for AZO exposure (Figures 6A–D). Similarly, the abnormalities of spindle assembly and chromosome alignment induced by AZO exposure were rescued by melatonin treatment (aberrant spindle, 65.58 ± 1.73 vs. 36.25 ± 6.70; chromosome misalignment, 78.08 ± 4.31 vs. 48.32 ± 3.59) (Figures 6E,F). In addition, our data showed that melatonin supplementation eliminated the excessive ROS and reversed mitochondrial dysfunction induced by AZO (1.39 ± 0.03 vs. 1.06 ± 0.049 for ROS intensity; 0.64 ± 0.03 vs. 0.78 ± 0.03 for mitochondrial intensity) (Figures 7A–E). Taken together, our data suggest that AZO induces mitochondrial dysfunction and excessive accumulation of ROS, while melatonin treatment effectively rescues oocytes from these impairments.

## Discussion

In recent years, there has been considerable interest in the effects of pesticides on human health. Azoxystrobin (AZO), a typical broad-spectrum fungicide, is one of the most frequently detected pesticide residues due to its widespread use (Park et al., 2022). The European Food Safety Authority (EFSA) has classified AZO as highly toxic to a majority of aquatic organisms (EFSA, 2010). For instance, AZO has been reported to have high reproductive toxicity in adult zebrafish (Cao et al., 2019). A recent study revealed that AZO exhibited reproductive toxicity in a dose- and time-dependent manner in male rats (El-Hak et al., 2022). However, the effect of AZO on mammalian oocyte maturation has not been well characterized. In this study, we found that AZO exposure impaired oocyte maturation not only by inducing mitochondrial dysfunction and oxidative stress, but also by decreasing MTOC integrity and subsequent spindle formation and chromosome alignment.

During oocyte maturation, GVBD and PBE are two critical markers of meiotic progression. Therefore, the effect of AZO on oocyte maturation was first examined by evaluating the GVBD and PBE rates. Previous studies have shown that AZO at 1 μM was sufficient to induce changes in transcriptional activity, while 5–10 μM could result in reactive oxygen species accumulation in primary neuronal cells (Pearson et al., 2016; Simon et al., 2019; Kang et al., 2021). Moreover, AZO at micromolar concentrations (1–3 μM) has been shown to modify membrane ion permeability in thymocytes (Imura et al., 2019). Accordingly, we chose a concentration range of 1–10 μM to test the effect of AZO on oocyte maturation. Our results showed that 5–10 µM AZO supplementation significantly reduced the GVBD and PBE rates, and 5 µM was an appropriate dose for further detection since this dose led to a considerable reduction in PBE rate. To further confirm the toxic effects of AZO on oocyte maturation, we examined spindle assembly and chromosome alignment, which are critical for meiotic progression. Aberrant spindle formation and lower intensity and irregular arrangement of chromosomes were frequently observed in AZO-exposed oocytes. Therefore, our results suggest that abnormalities in spindle assembly and chromosome alignment are the main contributors to impaired meiotic maturation after AZO treatment. However, despite the high incidence of spindle abnormalities, the PBE rate did not decrease proportionally. While the kinetochore microtubules directly attach to chromosomes and regulate chromosome segregation and, when impaired, activate spindle assembly checkpoint (SAC) and cause MI arrest, the polar microtubules do not interact with chromosomes but instead interdigitate with polar microtubules from the opposite pole, and thus are important to maintain spindle bipolarity and morphology (Wittmann et al., 2001). In this respect, we are tempting to speculate that the polar microtubules are more susceptible to AZO exposure than kinetochore microtubules. Thus, after AZO treatment, oocytes with aberrant spindle morphology may override SAC arrest and eventually undergo PBE. Given that spindle microtubules are emanated from MTOCs in mouse oocytes, it is also possible that decreased MTOC integrity may partially decrease spindle stability, which displays abnormal spindle morphology. Indeed, we observed that MTOC integrity was reduced after AZO treatment. Similar to our observation, it has been reported that a partial decrease in MTOC integrity caused severe spindle abnormalities, but partly disturbed PBE (Wang et al., 2017; Yin et al., 2021).

In contrast to somatic cells, mammalian oocytes lack canonical centrosomes because centrioles are lost during early oogenesis (Manandhar et al., 2005). Instead, multiple acentriolar microtubule organizing centers (MTOCs) functionally replace centrosomes and play a key role in oocyte microtubule nucleation (Schuh and Ellenberg, 2007; Clift and Schuh, 2015). Previous studies revealed that disruption of MTOC function in mouse oocytes could result in spindle instability, chromosome misalignment, and error-prone meiotic division that lead to female subfertility (Ou et al., 2010; Namgoong and Kim, 2018). Thus, we tested MTOC function by examining the levels of the MTOC markers CEP192, PCNT, and p-Aurora A (Lee et al., 2018). Our data showed that AZO treatment significantly decreased the number of MTOCs around chromosomes, as well as the intensity of CEP192, PCNT, and p-Aurora A, suggesting that impairments of spindle organization and chromosome alignment induced by AZO may be related to defects in MTOC assembly.

One basic mechanism of AZO cellular toxicity is its association with inhibition of the electron transfer chain in the oxidative phosphorylation process, impairing mitochondrial function (Shi et al., 2017). Mitochondria play an essential role in mammalian oocyte maturation by providing the ATP for meiotic division through oxidative phosphorylation. Notably, mitochondria surrounding the spindle periphery supply sufficient energy for spindle migration and chromosome segregation (Yu et al., 2010). Excessive clustering of mitochondria around the spindle, however, may lead to arrest of mouse oocytes at the metaphase I stage (Wang et al., 2020). Consistent with previous findings, our data showed that most mitochondria dispersed homogenously in the control oocyte cytoplasm, with enrichment around the spindle (Sathananthan and Trounson, 2000; Dalton and Carroll, 2013). However, the mitochondria in AZO-treated oocytes were heterogeneous and could not form a regular mitochondrial ring around the spindle, suggesting that AZO supplementation disrupted the distribution of cytoplasmic mitochondria. Indeed, accumulation of mitochondria around the spindle was associated with migrating cytoplasmic MTOCs, driven by dynein, during the first mitotic division in mouse oocytes (Dalton and Carroll, 2013). Our data showed that AZO treatment significantly compromised the integrity of MTOCs. Therefore, we propose that the mitochondrial dysfunction induced by AZO contributed to the failure of spindle assembly and migration, leading to PBE failure during meiotic maturation in mouse oocytes.

Typically, dysfunctional mitochondria are less able to counteract ROS production, which contributes to oxidative stress. We therefore examined the ROS levels in oocytes exposed to AZO. As expected, AZO addition caused excessive accumulation of ROS. Previous studies suggested that oxidative stress had negative impacts on oocyte maturation and embryo development, while the application of antioxidants could be effective in rescuing the defects of oocyte maturation (Cajas et al., 2020; Cao et al., 2021). Melatonin, a hormone produced by many organs including the pineal gland, has strong free radical scavenging and antioxidant properties and has been found to protect oocytes from oxidative damage (Pang et al., 2016; Xing et al., 2021). Mechanistically, melatonin protects against oxidative stress mainly through inducing the expression of antioxidant enzymes, inactivating pro-oxidants, and maintaining mitochondrial homeostasis (Zhang and Zhang, 2014). Accordingly, we used melatonin to determine whether defects in oocyte maturation could be eliminated during AZO exposure. Our data showed that defects in chromosome organization, spindle and MTOC assembly, and first PBE after AZO treatment were remarkably restored by melatonin during mouse oocyte maturation. Moreover, excessive ROS production and heterogeneous mitochondrial distribution induced by AZO were significantly reduced by melatonin addition. Given that the expression of antioxidant enzymes decreased after AZO exposure, it is likely that melatonin may restore the expression of these enzymes and thereby improve ROS scavenging capacity of oocytes. Consistent with our results, melatonin has been reported to improve oocyte quality by decreasing ROS levels (Jiang et al., 2021). However, the precise mechanisms of melatonin in protecting oocytes against AZO-induced toxicity need to be determined.

In conclusion, our results demonstrate that AZO exposure results in meiotic arrest by disrupting MTOC-mediated spindle assembly and chromosome alignment, mainly due to dysfunctional mitochondria and excessive ROS accumulation induced by AZO. Moreover, melatonin, due to its antioxidant properties, is likely to be a promising compound to rescue oocyte maturation from AZO-induced cytotoxicity. Given that *in vitro* assay may not replicate the precise cellular conditions of an organisms, we could not entirely exclude that AZO could react with biological components after entering into circulation system in the body and generate secondary metabolites, which in turn indirectly affects cellular system including oocyte maturation. It is also considerable the compensatory effects from surrounding environment such as follicular fluids and granulosa and cumulus cells in the ovary. Therefore, further verification of cytotoxicity of AZO on oocyte maturation is required using *in vivo* model system.

## Data Availability

The original contributions presented in the study are included in the article/supplementary material, further inquiries can be directed to the corresponding author.
